# Exploratory analysis and framework for tissue classification based on vibroacoustic signals from needle–tissue interaction

**DOI:** 10.1007/s11548-025-03491-1

**Published:** 2025-08-12

**Authors:** Katarzyna Heryan, Witold Serwatka, Dominik Rzepka, Patricio Fuentealba, Michael Friebe

**Affiliations:** 1 Institute of Computer Science, AGH University of Kraków, al. Adama Mickiewicza 30, 30-059 Kraków, Poland; 2 Instituto de Electricidad y Electrónica, Facultad de Ciencias de la Ingeniería, Universidad Austral de Chile, 5111187 Valdivia, Chile

**Keywords:** Vibroacoustic signals, Denoising algorithm, Signal processing, Convolutional neural networks, Needle guidance, Interventional procedures, Minimal invasive therapies

## Abstract

**Purpose:**

Numerous medical procedures, such as pharmaceutical fluid injections and biopsies, require the use of a surgical needle. During such procedures, the localization of the needle is of prime importance, both to ensure that no vital organs will be or have been damaged and to confirm that the target location has been reached. The guidance to a target and its localization is done using different imaging devices, such as MRI machines, CT scans, and US devices. All of them suffer from artifacts, making the accurate localization, especially the tip, of the needle difficult. This implies the necessity for a new needle guidance technique.

**Methods:**

The movement of a needle through human tissue produces vibroacoustic signals which may be leveraged to retrieve information on the needle’s location using data processing and deep learning techniques. We have constructed a specialized phantom with animal tissue submerged in gelatine to gather the data needed to prove this hypothesis.

**Results and conclusion:**

This paper summarizes our initial experiments, in which we preprocessed the data, converted it into two different spectrogram representations (Mel and continuous wavelet transform spectrograms), and used them as input for two different deep learning models: NeedleNet and ResNet-34. The goal of this work was to chart out an optimal direction for further research.

## Introduction

Interventional procedures, minimal-invasive therapies, and image-guided therapies (IGT) have become pivotal components of modern medical practice, offering less invasive alternatives to traditional surgical approaches. Central to these advancements are medical interventional devices (MIDs) such as aspiration and biopsy needles, which enable clinicians to perform a wide range of clinical procedures with precision and minimal patient discomfort [1, 2]. These devices find application in diverse medical contexts, from diagnostic biopsies to therapeutic interventions [1, 3, 4].

In certain clinical scenarios, these MID are used without external imaging guidance, particularly when the target site is close to the skin, devoid of sensitive structures along the needle path, and where precise placement might be less critical [1, 2]. An instance of this scenario is when a pharmaceutical solution is intended to diffuse into adjacent tissue, obviating the need for high-level placement accuracy.

On the contrary, there are cases where precise placement is critical to ensure accurate MID path traversal, when it is important to get a confirmation on having reached the target site, and to verify the procedure efficacy. In such cases, external imaging guidance becomes indispensable [1–3].

While imaging methods such as ultrasound, X-ray, CT, and MRI are employed for guidance, they can introduce artifacts that obscure needle location and accurate tissue targeting [5]. These challenges are particularly evident in procedures requiring precise needle placement and confirmation of target site attainment. However, imaging modalities–especially MRI–offer the crucial advantage of visualizing not only the needle trajectory but also its spatial relationships with surrounding anatomical structures. The value of such imaging for precision in robotic and image-guided surgery is exemplified by Dallan et al. [6]).

Ultrasound (US), planar X-ray, Computed Tomography (CT), and in specific cases, Magnetic Resonance Imaging (MRI), serve as the primary imaging modalities for guiding these procedures [7–9].

While US holds wide applicability as the system of choice for most of the simpler applications and is harmless in terms of radiation exposure, X-ray and CT expose both, clinicians and patients, to harmful radiation, and MRI requires the use of specialized, compatible, and costly MID to avoid magnetic attraction forces and to ensure high image quality. Nonetheless, all these imaging techniques exhibit limitations, often resulting in artifacts that obscure the MID’s path, diameter, and precise location within the anatomy. This becomes particularly pertinent when the MID tip remains partially or entirely outside the imaging field of view [7, 9, 10]. To address these issues, there is a growing interest in adding sensors to enhance the guidance during these procedures.

To enhance the guidance and sensing capabilities of existing MIDs adding force or other contact sensors directly to the needle tip has been proposed, but this approach has many drawbacks including reduced device functionality, complex and with that expensive construction, typically the need for an active cable connection, and a complicated operation and setup [5]. A more favorable approach involves developing proximally attached clip-on systems, based on measuring vibroacoustic signals generated in the interaction between the moving MID and the tissue that the MID penetrates through [11–14]. These generated waves propagate through the MID shaft to its proximal end, where they can be captured as audio signals for subsequent processing and analysis [15]. Accurate determination and classification of these signals for in vivo applications demands robust testing, dedicated in vitro and ex-vivo experimental setups with specialized tissue phantoms that replicate real tissue interactions [11–14].

Our previous works [12, 16] have demonstrated the potential of vibroacoustic signals for classifying MID related penetration events during catheter and needle procedures.

In this paper, we introduce a dedicated framework combining signal processing and deep learning for tissue classification during needle procedures. Leveraging these audio signals in combination with deep learning techniques for tissue classification could enhance real-time guidance during medical interventions.

The primary research objective and goal of this paper is to investigate the feasibility and effectiveness of utilizing vibroacoustic signals captured through audio sensors for tissue classification during needle procedures in an experimental setup using dedicated phantoms. It was our aim to demonstrate that these signals, combined with advanced signal processing techniques and deep learning algorithms, can reliably differentiate between different artificial and ex-vivo tissue types. If accurate tissue classification could be achieved in the experimental setup it might be a feasible technology to enhance the guidance and confidence of performing needle procedures.

The goal of this paper is to present a novel approach to tissue classification based on vibroacoustic signals captured from needle–tissue interaction with following key contributions:

Novel Signal Analysis: Introduce a dedicated denoising algorithm inspired by ECG signal processing to enhance the quality of vibroacoustic signals captured during needle–tissue interaction.

Signal Representation Exploration: Explore and compare different signal representations, including Continuous Wavelet Transform (CWT) and Mel Scale, to identify the most effective representation for tissue classification.

Deep Learning Architectures: Propose and evaluate two deep learning architectures, namely Needle Net and ResNet, for accurate tissue classification using the processed audio signals.

Validation: Experimental results demonstrate that the proposed strategy leads to promising classification results for different tissue types, paving the way for future research to enhance guidance during needle procedures.

## Data gathering and preprocessing

**Fig. 1:**
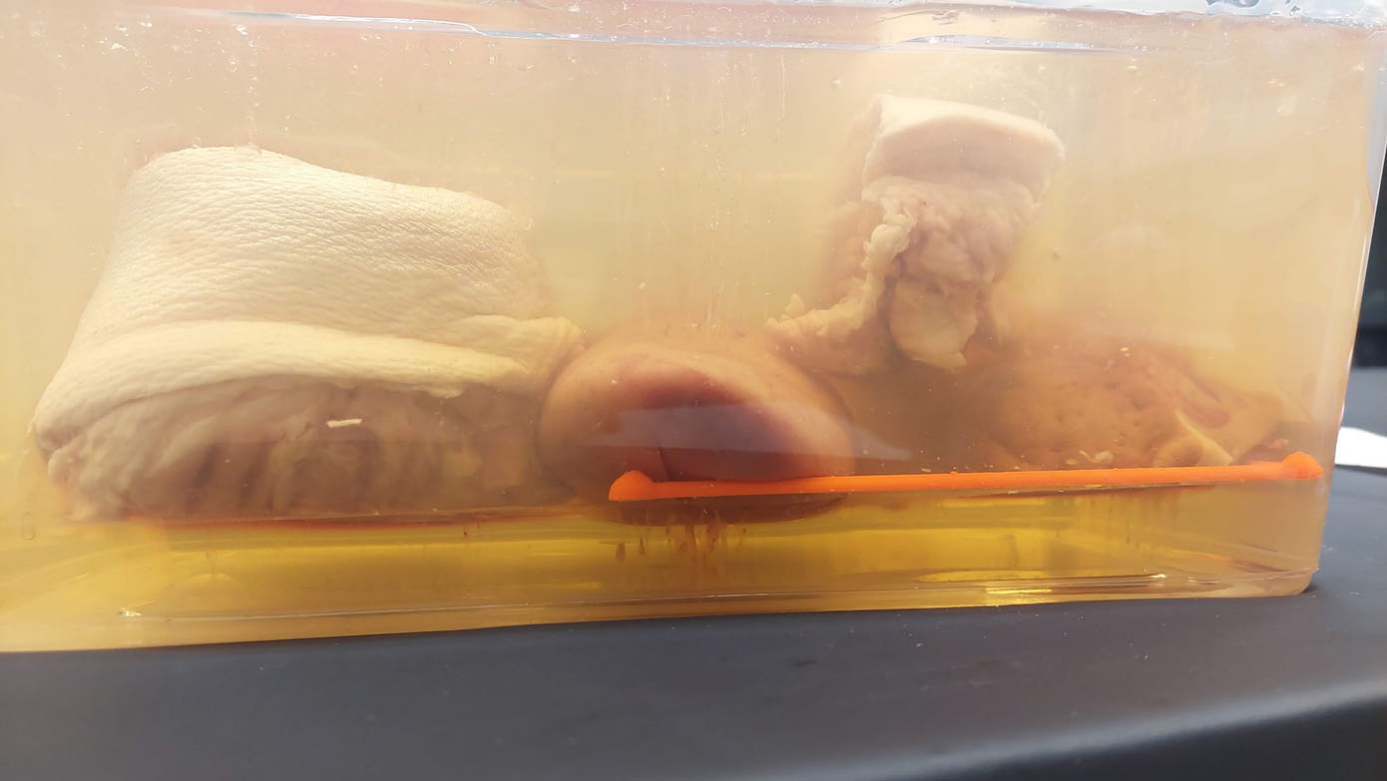
Animal phantom: from the left side: knee, kidney, ribs, and liver, respectively

**Fig. 2:**
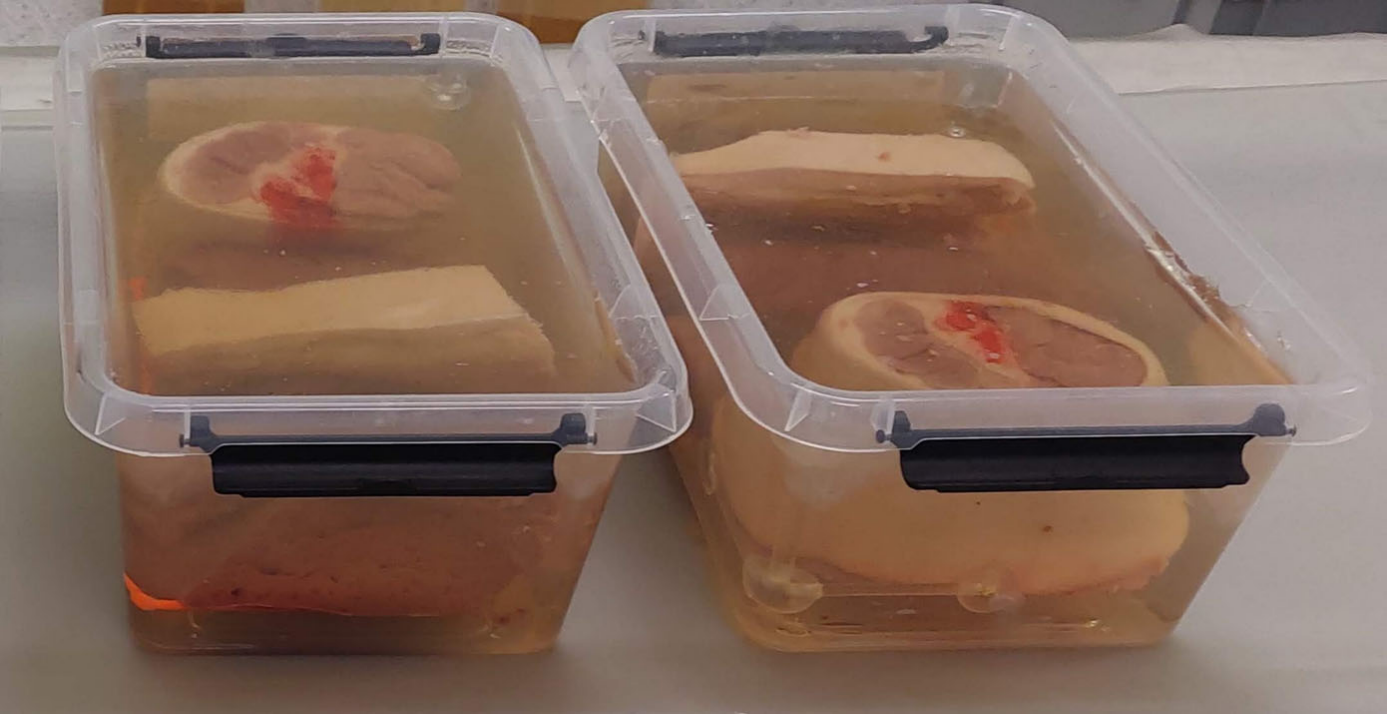
Two similar animal phantoms with the same localization of particular animal parts

To facilitate the initial experimental data collection, a dedicated phantom was designed. The phantom consisted of animal organs such as the liver, kidney, rib, muscle, and skin, which were immersed in a gelatine medium. The phantom’s assembly followed a simple process: a semi-hardened gelatine layer served as the base upon which the anatomical structures were positioned. Additional gelatine was then carefully poured to ensure complete submersion of all items. Two similar phantoms, containing pork animal tissues , were prepared (Fig. 2). The placement of materials was consistent across both phantoms, ensuring replicability of the experimental procedure. Geometry determinants, depicted in (Fig. 1) were also incorporated into each phantom, with one set produced using an MK3S+ Prusa printer and PLA material, and the other set using an Anycubic Photon Mono X 6K printer and Water-wash resin. These geometry determinants were essential to guarantee the reproducibility of the phantoms’ configurations, thus enhancing the reliability of future research involving 3D image data analysis (Fig. 2).

For filling, a technical gelatine with characteristics including high purity, platinum content, and a Bloom value of 240, was employed. The extracted material classifications encompassed the liver, kidney, knee region skin, knee region muscle, and the intricate multilayered composition from the rib region.

The experiments were conducted using a specialized audio acquisition module (developed by SURAG GmbH, Magdeburg, Germany), that included a MEMS microphone and wireless data transfer capability. This module was linked to a commercially available aspiration needle provided by I.T.P. GmbH, Bochum, Germany, through a luer-lock connection.

**Fig. 3:**
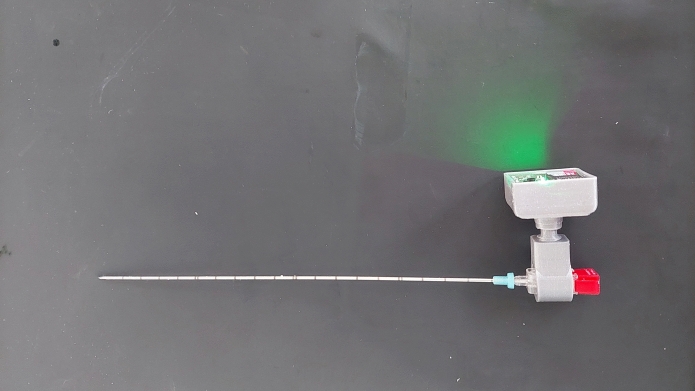
The sensing device used in the experiment, consists of an audio acquisition module and an aspiration need

During the experimental measurements, the MEMS microphone of the audio acquisition module was positioned external to the phantom. The audio signals generated during the experiments were transmitted through the needle’s shaft and captured by an external recording device. To minimize the impact of ambient noise, both hardware-based ambient noise reduction techniques and a mechanical cover were employed (refer to Fig. 3). The specifics of the audio acquisition device used in this setup can be found in [17].

The data collection process is initiated when the aspiration needle comes into contact with the gelatin surface. Recordings begin at this point and continue until the needle punctures and traversals through the phantom material.

In total, 1282 audio samples were considered suitable for analysis, all of which were sampled at a rate of 22050Hz. The dataset included an almost equal distribution of samples from each class:

- Class 0 (idx kidney): 231 instances,
- Class 1 (idx liver): 300 instances,
- Class 2 (idx muscle): 208 instances,
- Class 3 (idx rib): 211 instances,
- Class 4 (idx skin): 332 instances.

**Fig. 4:**
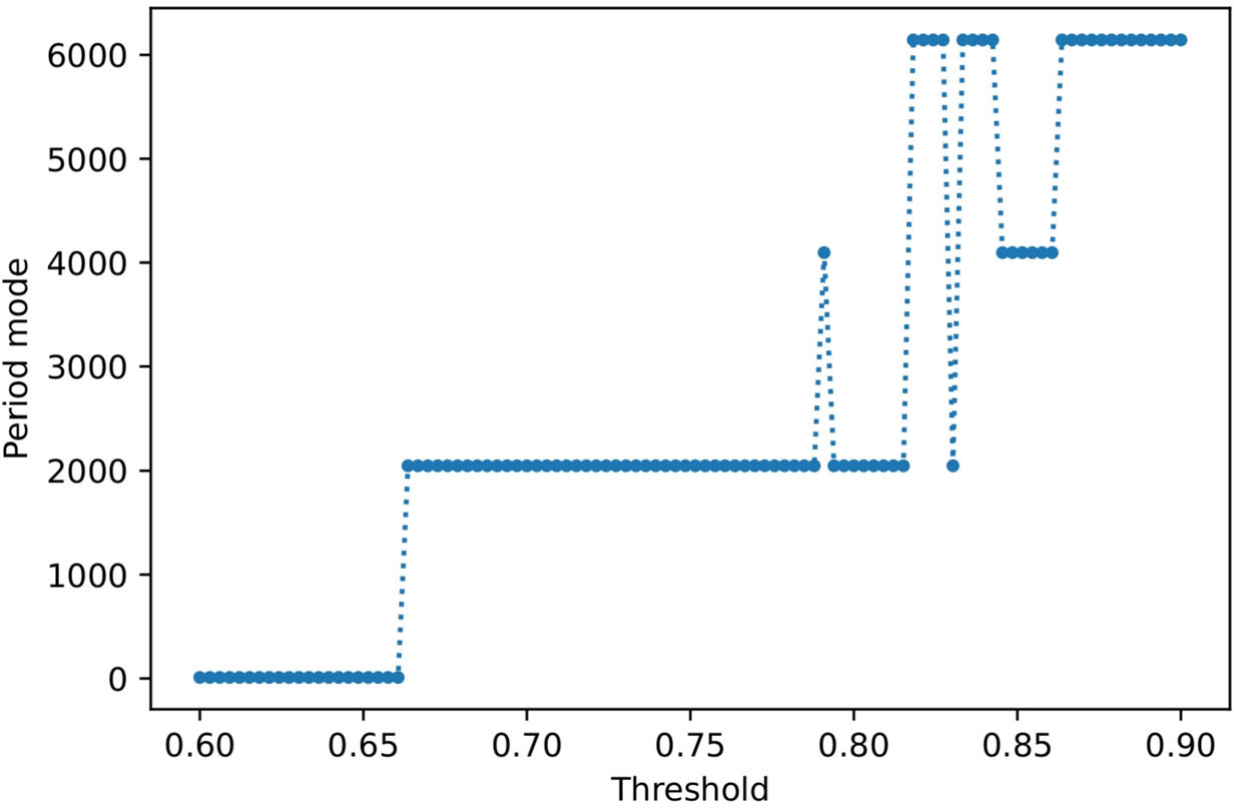
Mode of the distance between upcrossings of signal for a given threshold

The raw audio signals turned out to be heavily corrupted by impulsive periodic noise, likely produced by some device in the laboratory room and recorded as a result of the high sensitivity of the microphone. Due to the impulsive nature of the unwanted component and its relatively long period, classical digital filtering methods and frequency-domain analysis were not effective in its removal. However, it was possible to identify and filter out the interfering pulse train using a time-domain method based on the template subtraction method commonly used in ECG processing [18, 19]. To find the period of the pulse train, the distance between consecutive spikes was measured by observing the difference $T_n=t_n-t_{n-1}$ between consecutive upcrossings of the threshold value $\theta$ by a measured signal, previously debiased and normalized to [$-1$, 1] range. To avoid choosing some ad-hoc value of the threshold, the procedure was performed for the range $\theta \in (0.6, 0.9)$. For each value of $\theta$, the mode of distances $T_n$ was computed. Results presented in Fig. 4 show that for threshold value large enough to discriminate spikes of the interfering pulse train, dominant distance between spikes is equal to ${\hat{T}}=2048$ samples. This value was also obtained for other recordings. For larger values of the threshold, the dominant distance becomes multiplicity of ${\hat{T}}$.

**Fig. 5:**
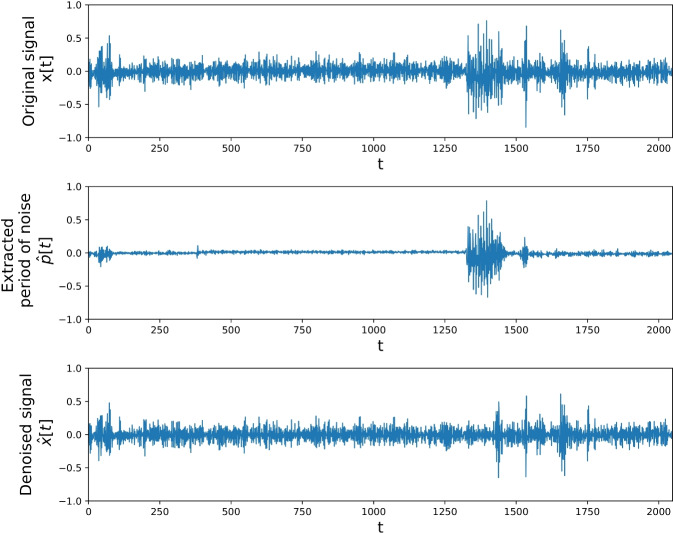
Example of signal denoising: (top) Original signal; (middle) estimated pulse signal; (bottom) denoised signal

The shape and amplitude of the periodic pattern were observed to be similar across each recording. Given the fact that the signal was debiased, and assuming that the vibroacoustic signal is stationary and independent of the corrupting signal, the template of pulse signal ${\hat{p}}[\tau ]$ could be extracted by averaging ${\hat{T}}$-samples chunks of the original signal, $x[t], t\in n{\hat{T}}, \ldots , (n+1){\hat{T}}$. However, as the useful signal is not stationary, the more robust method is to use the median of the signal values instead of the mean,

1 $$\begin{aligned} {\hat{p}}[\tau ]=\textrm{Median}(x[\tau ], x[\tau +{\hat{T}},\ldots , x[\tau +N{\hat{T}}), \end{aligned}$$

where $N{\hat{T}}$ does not exceed the length of the recording. Finally, to obtain a denoised signal, the periodic signal

2 $$\begin{aligned} {\hat{p}}[t] = {\hat{p}}[\tau ], \tau = t\;\textrm{modulo}\;{\hat{T}} \end{aligned}$$

is created from the estimated pulse signal ${\hat{p}}[\tau ]$, and subtracted from the source signal ${\hat{x}}[t]=x[t]-{\hat{p}}[t].$ The example of signal denoising is presented in Fig. 5.

In order to create the training datasets, raw and denoised audio signals were processed identically with the goal to investigate the effectiveness of the denoising algorithm, as well as to compare two spectrogram representations of the data: Mel spectrograms and continuous wavelet transform (CWT).

**Fig. 6:**
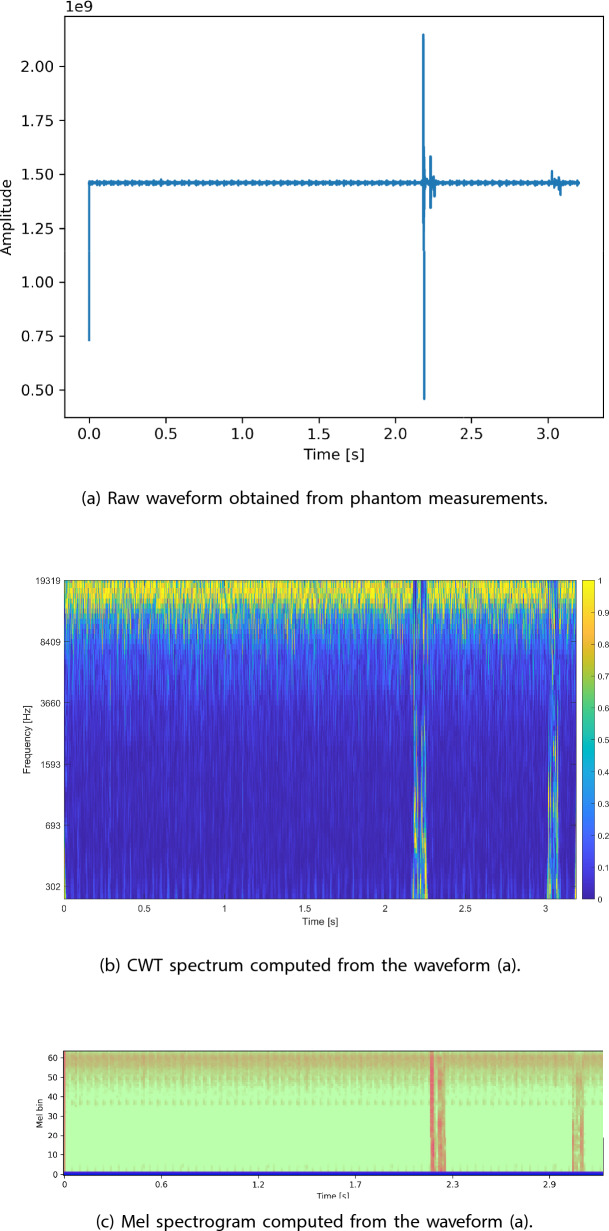
An exemplary waveform obtained from measurements conducted on the phantoms. This raw signal was then converted into a Mel spectrogram and a CWT spectrogram in order to compare both representations with each other

**Fig. 7:**
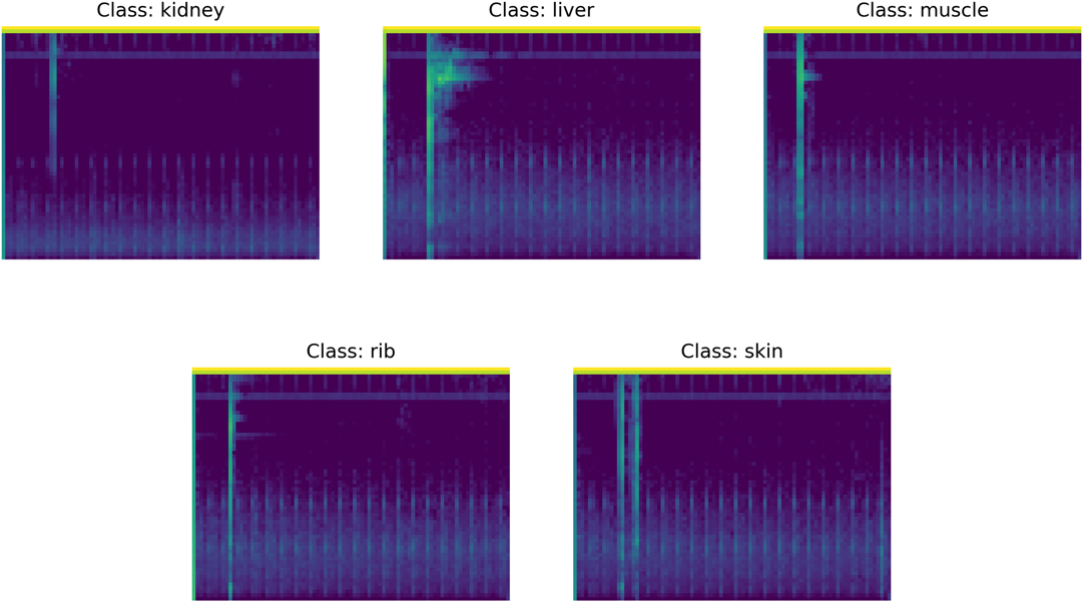
Mel spectrogram representations of data points belonging to the five tissue types

The former representation was created by applying the short-time Fourier transform on the audio signals. The transform used 1024 windows with a hop length of 512. The result was converted into a Mel spectrogram with 64 Mel bins. As a final step, the amplitude spectrograms were converted to dB spectrograms. An example Mel spectrogram is presented in Fig. 6b, Mel spectrograms representing different classes are presented in Fig. 7.

The second representation was obtained by applying the CWT on the audio signal. The time-scale CWT spectral representation was computed using a perfectly symmetric Morse mother wavelet with a time-bandwidth product equal to 60. An example of the obtained CWT spectrum is shown in Fig. 6c, which was computed from the example signal presented in Fig. 6a. In the presented figure, the x-axis corresponds to time in seconds, and the y-axis corresponds to the frequency in Hz. The spectral energy is represented by a color map, where the blue indicates a lower energy and the yellow indicates a higher energy level.

## Models and training

**Fig. 8:**
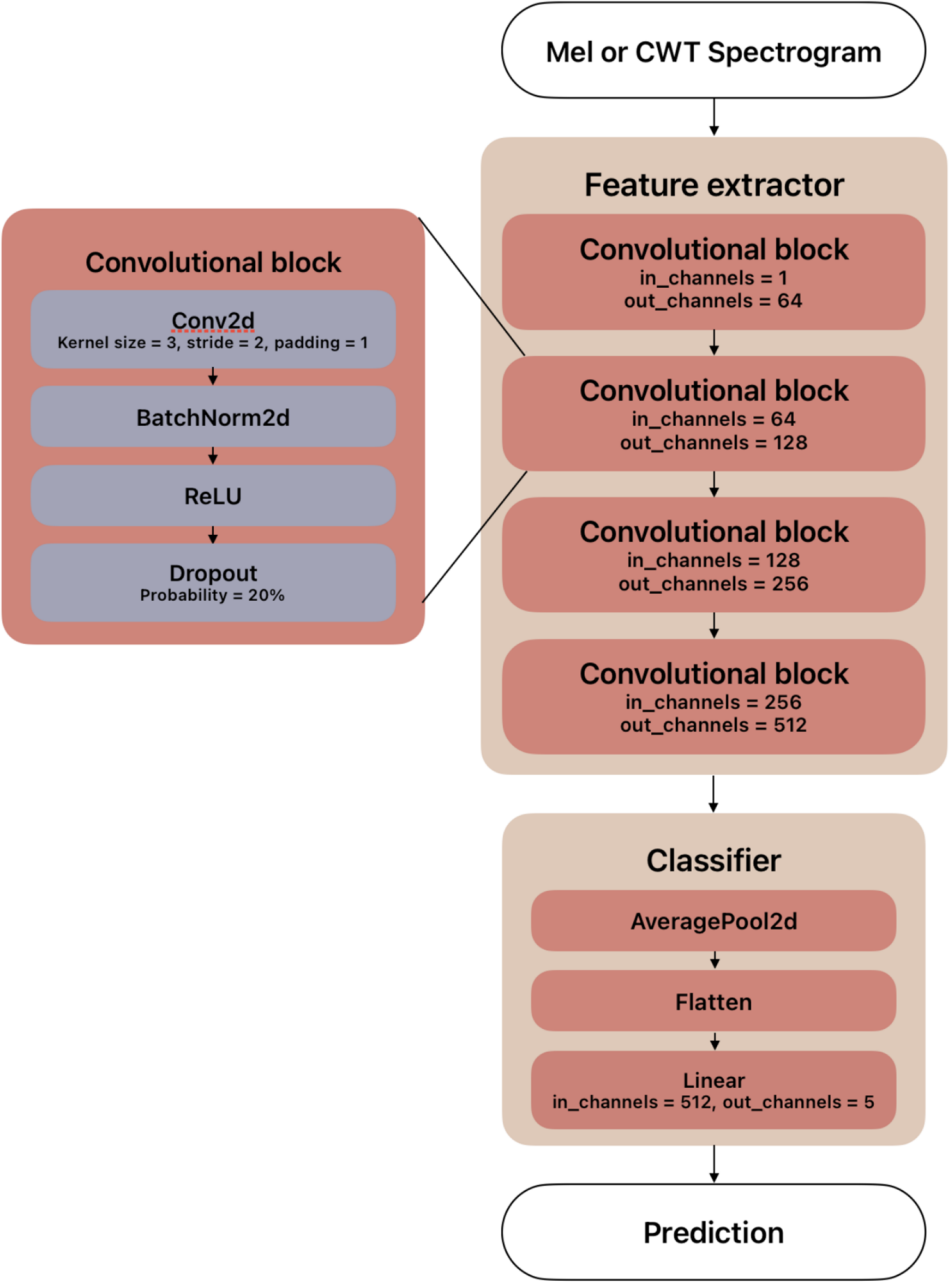
The architecture of NeedleNet, a CNN created to provide a baseline for further experiments

For the tissue classification task two models were selected. The first one is called NeedleNet, a convolutional neural network (CNN) created for the sole purpose of this research. It consists of four convolutional blocks, with each block containing a two-dimensional convolutional layer with a ReLU activation, a dropout layer and a two-dimensional batch normalization layer. The convolutional blocks have 64, 128, 256 and 512 convolutional filters, respectively. The convolutional layers have a kernel size of 3, a stride of size 2 and a padding equal to 1. The dropout probability was set to .2. The convolutional blocks are followed by an adaptive average pooling layer and one fully connected layer. It is worth noting that the adaptive pooling layer allows us to input spectrograms of different sizes into the model, without the need of resizing them. Because of that, we can easily compare the model’s performance, given different data representations. On the downside, the model is not fine-tuned to a specific input size, which can result in poor performance. A detailed schema explaining the design of NeedleNet is presented in Fig. 8.

The second model used in our experiments is ResNet [20]. This network was chosen due to it’s established effectiveness in different machine learning tasks, including acoustic signal classification in a medical setting [21]. In this research, we have specifically used a 34-layer, pre-trained variant, available in the PyTorch library [22]. We have made use of previous research [23] that showed improved results in audio classification when pre-trained models were employed, even when they were trained on real-world image datasets. In this case, our ResNet was trained on ImageNet before being used for our research. The last layer of the model was accommodated to differentiate between 5 classes, in contrast to the default 1000. Since the architecture of ResNet requires the dimensions of the input image to be at most 224 pixels, the CWT spectrograms are resized to 224 by 160 pixels. This specific size was chosen in order to keep the ratio of the original and resized spectrograms the same.

Both models were trained and evaluated on the prepared datasets. The training environment and setting was identically for each model-dataset pair. Every pair was trained for 150 epochs with a learning rate of 0.01, a momentum of .9 and a batch size of 32. The optimization method was stochastic gradient descent and the loss function was cross-entropy. A 5-fold crossvalidation was used to achieve more accurate results.

This meant that for every fold approximately 256 out of the total 1282 were set aside for testing. A higher number of folds would result in unreliable, fluctuating training metrics values.

**Fig. 9:**
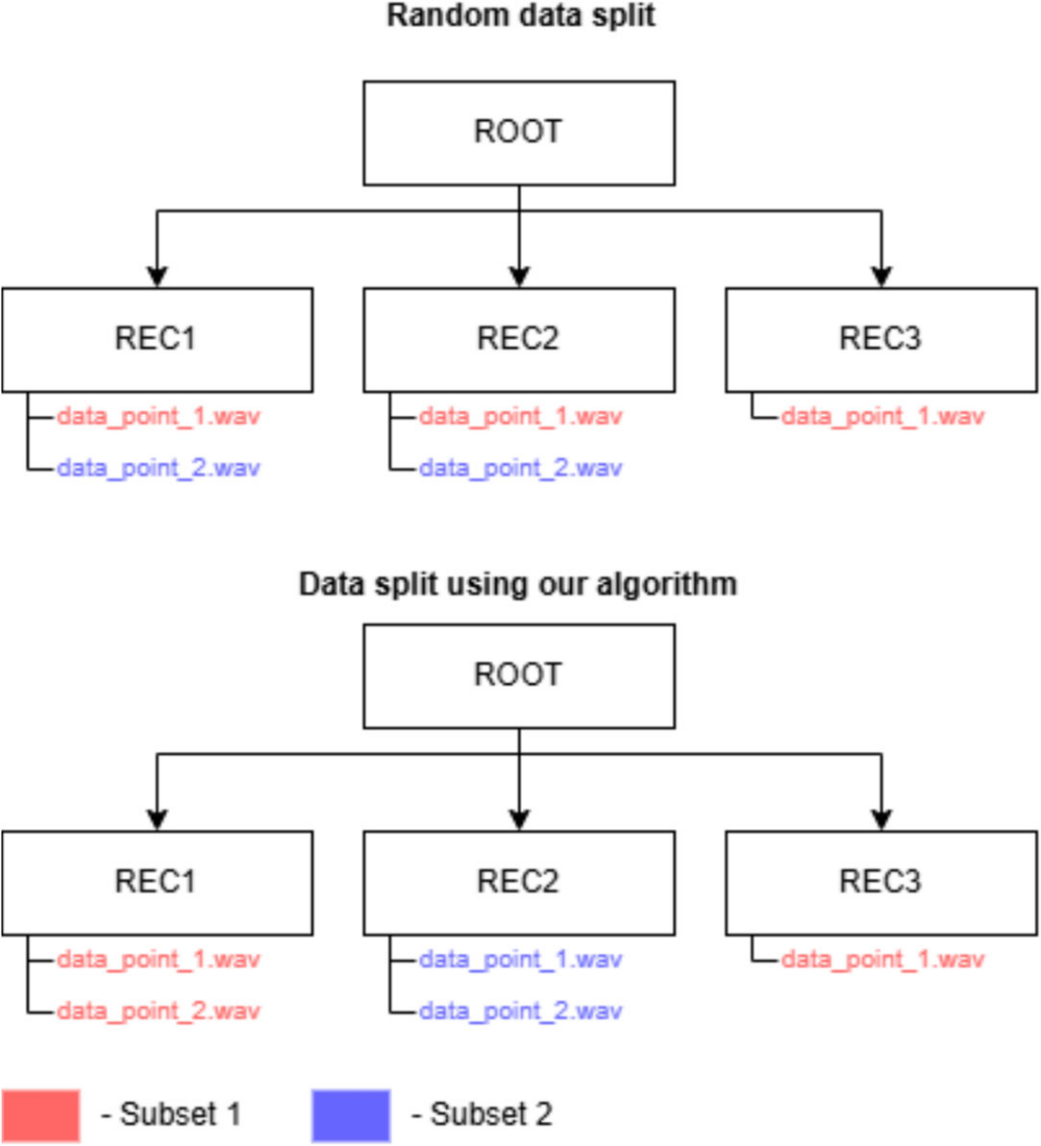
Two ways to split the datasets: on the left, the more commonly used random data split results in data points from the same recording to be split into two different subsets. Our algorithm, shown on the right, makes sure that all data points from one recording are part of one subset

In order to prevent data leakage, we implemented a custom data split algorithm, which groups the data points by the original recording they belonged to originally. First, the algorithm counts the number of data points per recording, as well as the number of data points per class. Next, it calculates the number and classes of data points that should be included in the splits. The subsets are created by selecting a recording name by random and assigning it to the split. The number of data points extracted from the recording is subtracted from the number of data points required for the split, until no more samples are required. Figure 9 shows an example of how this algorithm splits the data, compared to the more commonly used random data split.

## Results

**Fig. 10:**
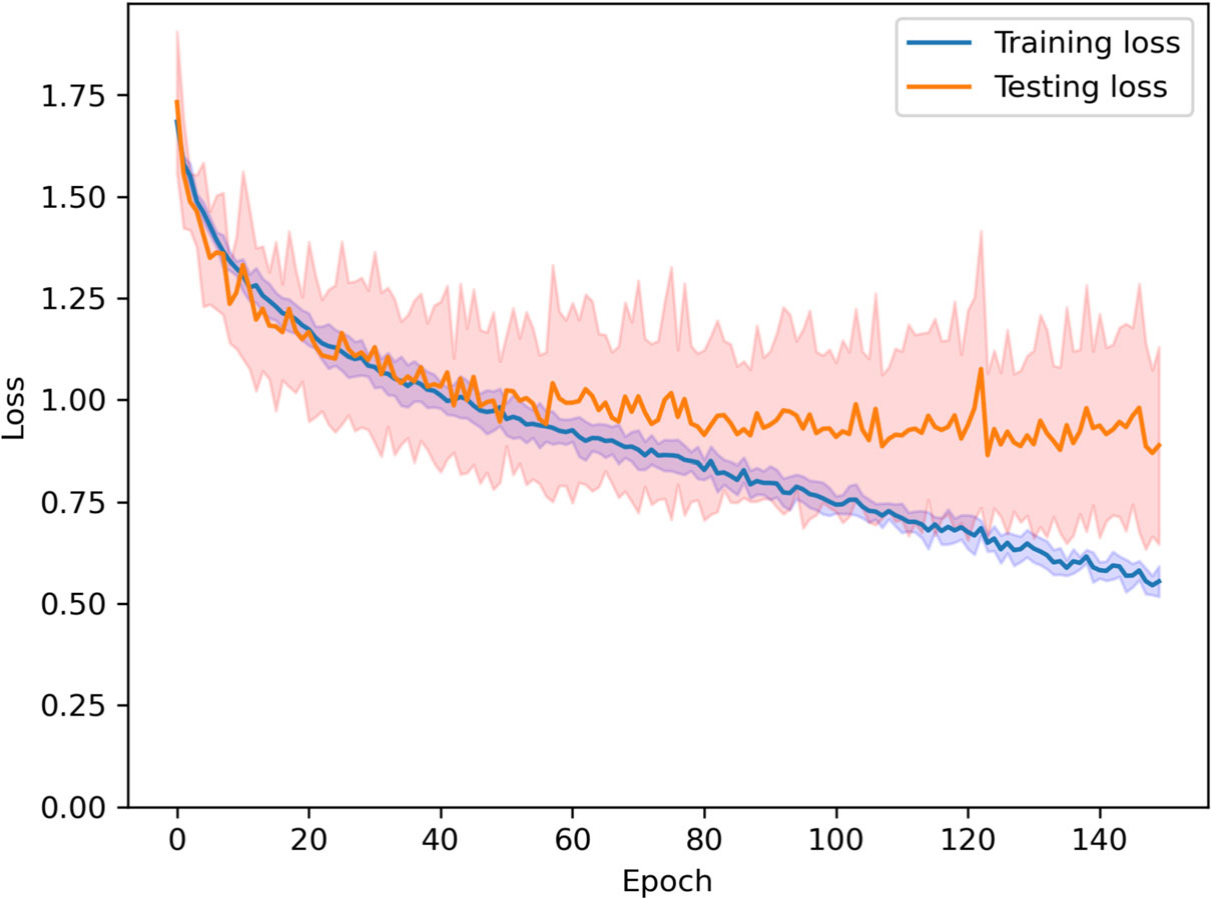
Training and testing losses for one of the training runs on NeedleNet, with a 95% confidence interval

The training parameters were chosen carefully in order for the models to slightly overfit. This was done in order to better investigate their performances and make sure that they achieved their full training capacity on the models. Figure 10 shows an exemplary loss progression for the training and testing phases of NeedleNet on one of the datasets. The learning curves of all model-dataset pairs were very similar. Since we employed crossvalidation, we can show here the 95% confidence rate of the loss values.

**Fig. 11:**
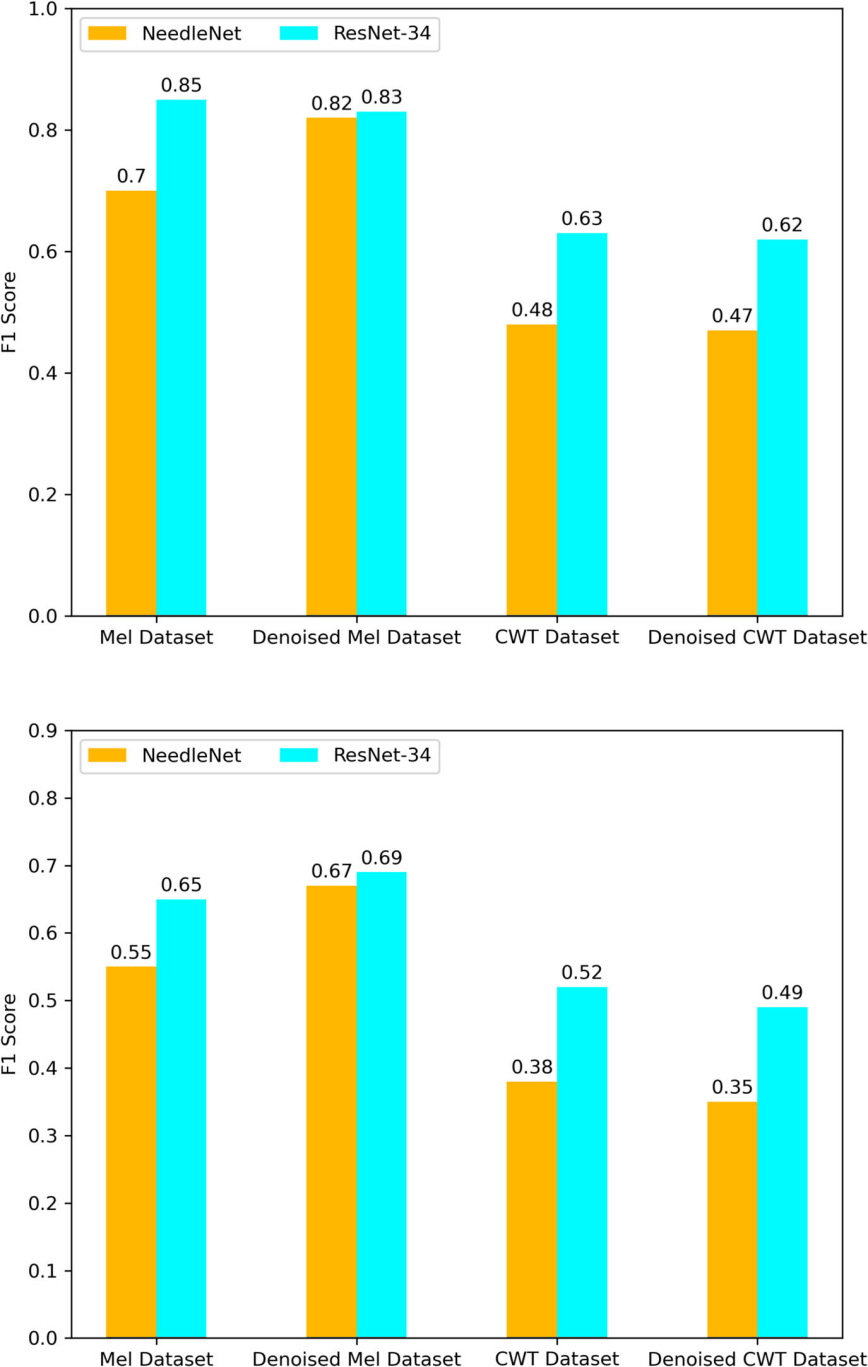
Best and average F1 scores achieved during the training of NeedleNet and ResNet-34 on different datasets, using 5-fold crossvalidation

During the training, we measured the effectiveness of the model using accuracy, precision and F1 score metrics. The model’s performances were measured after every epoch of the training. To summarize the results of the crossvalidation, we decided to save the best observed result during all folds, as well as the average final metrics values of the folds. Figure 11 shows these results for the F1 score in detail for both the models and every dataset they were trained on. Table 1 contains all training results, including accuracy, precision and F1 score metrics, their best and average values.

**Table 1:** Best accuracy, precision and F1 score values achieved during training, with average values included inside parentheses

Configuration	Accuracy	Precision	F1 Score
NeedleNet on	70.33%	76.52%	70.05%
Mel dataset	(56.91%)	(59.34%)	(55.07%)
ResNet-34 on	86.08%	85.45%	85.03%
Mel dataset	(67.96%)	(68.05%)	(64.92%)
NeedleNet on	82.99%	81.88%	81.54%
denoised Mel dataset	(67.68%)	(69.86%)	(67.05%)
ResNet-34 on	83.81%	84.85%	82.92%
denoised Mel dataset	(71.31%)	(71.71%)	(69.5%)
NeedleNet on	52.47%	54.86%	48.2%
CWT dataset	(42.44%)	(41.77%)	(37.87%)
ResNet-34 on	65.71%	69.06%	63.37%
CWT dataset	(55.05%)	(55.9%)	(52.91%)
NeedleNet on	50.49%	52.25%	46.51%
denoised CWT dataset	(38.95%)	(39.86%)	(35.17%)
ResNet-34 on	61.76%	65.92%	61.63%
denoised CWT dataset	(50.73%)	(51.93%)	(49.35%)

## Discussion

This study demonstrates the feasibility of using vibroacoustic signals from needle–tissue interactions for tissue classification in an ex-vivo setting using deep learning. Results from both the custom NeedleNet and the pre-trained ResNet-34 models confirm that the spectral characteristics of these signals are sufficient to distinguish between the five studied tissue types.

### Comparison with existing scientific literature

The findings are consistent with prior work employing audio-based sensing for instrument tracking and event detection in minimally invasive procedures. Illanes et al. [12] and Schaufler et al. [14] demonstrated that audio features enable the detection of procedural events such as tissue puncture, albeit in binary classification settings. This study extends previous efforts by exploring multi-class tissue classification and implementing a complete framework with denoising, spectrogram transformation, and model comparison.

The superior performance of ResNet-34 on denoised Mel spectrograms (accuracy 83.81%, F1 score 82.92%) aligns with previous findings on transfer learning for audio classification and validates the utility of pre-trained architectures for this application.

### Clinical relevance and integration potential

Accurate needle placement is essential in procedures such as biopsies, regional anesthesia, and central line insertion. Conventional imaging techniques (e.g., ultrasound, CT, MRI) offer visualization but are often limited by artifacts or lack of real-time feedback near the needle tip [24]. Vibroacoustic sensing could offer complementary information on tissue identity, enhancing safety and precision.

A practical implementation may involve a sterilizable clip-on sensor that captures real-time audio signals, connected to a low-latency classification system. Such integration could improve decision-making during procedures by signaling transitions between tissue types and avoiding critical structures.

### Integration into hospital workflows

To integrate the proposed system into clinical environments, several factors require consideration:

- Sterilizable, wireless sensor design.
- Compatibility with hospital IT infrastructure (e.g., PACS).
- Real-time signal processing on embedded or mobile platforms.
- Robustness across various needle types and environmental conditions.
- User interfaces that deliver feedback without distracting clinicians.

Initial adoption may be most feasible in educational or low-risk diagnostic procedures.

### Limitations and future work

This study used ex-vivo animal tissue in a controlled laboratory setup, which does not replicate in vivo physiological conditions such as perfusion, motion, and thermal variability. The dataset, while carefully annotated, is also limited in size.

Generalization of deep learning models remains a challenge. Future research should address:

- Expansion to human cadaver and in vivo datasets.
- Evaluation under diverse clinical and environmental conditions.
- Hardware miniaturization for clinical compatibility.
- Fusion with other modalities (e.g., force feedback or imaging).

These steps are essential for transitioning from proof-of-concept to clinical deployment.

## Conclusion

To validate this approach, dedicated phantoms containing animal tissues immersed in gelatine were constructed to emulate realistic needle–tissue interactions. Vibroacoustic signals were recorded using an audio acquisition module attached to a commercial aspiration needle. Signal quality was enhanced through noise reduction techniques and mechanical shielding.

The acquired signals were processed using advanced denoising algorithms and converted into Mel and continuous wavelet transform (CWT) spectrograms. These representations were used to train two deep learning models–NeedleNet and a pre-trained ResNet-34. Both models demonstrated promising initial classification performance, supporting the feasibility of tissue identification based solely on acoustic data.

This work introduces a novel framework for audio-based tissue classification and highlights its potential as a real-time guidance tool for minimally invasive procedures. With further validation and integration, this approach could support safer and more accurate needle placement in clinical practice.

## References

[1] Abolhassani N, Patel R, Moallem M (2007) Needle insertion into soft tissue: a survey. Med Eng Phys 29(4):413–431. 10.1016/j.medengphy.2006.07.003 16938481 10.1016/j.medengphy.2006.07.003

[2] van Gerwen DJ, Dankelman J, van den Dobbelsteen JJ (2012) Needle–tissue interaction forces: a survey of experimental data. Med Eng Phys 34(6):665–680. 10.1016/j.medengphy.2012.04.007 22621782 10.1016/j.medengphy.2012.04.007

[3] Okamura AM, Simone C, O’Leary MD (2004) Force modeling for needle insertion into soft tissue. IEEE Trans Biomed Eng 51(10):1707–1716. 10.1109/TBME.2004.831542 15490818 10.1109/TBME.2004.831542

[4] Trejos AL, Patel RV, Naish MD (2010) Force sensing and its application in minimally invasive surgery and therapy: a survey. Proc Inst Mech Eng C J Mech Eng Sci 224(7):1435–1454. 10.1243/09544062JMES1917

[5] Abayazid M, Kemp M, Misra S (2013) 3d flexible needle steering in soft-tissue phantoms using fiber bragg grating sensors, pp 5843–5849. 10.1109/ICRA.2013.6631418

[6] Dallan I, Seccia V, Faggioni L, Castelnuovo P, Montevecchi F, Casani AP, Tschabitscher M, Vicini C (2013) Anatomical landmarks for transoral robotic tongue base surgery: comparison between endoscopic, external and radiological perspectives. Surg Radiol Anat 35(1):3–10. 10.1007/s00276-012-0983-2 22644779 10.1007/s00276-012-0983-2

[7] McWilliams S, Murphy K, Golestaneh S, O’Regan K, Arellano R, Maher M, O’Connor O (2014) Reduction of guide needle streak artifact in ct-guided biopsy. J Vasc Intervent Radiol JVIR. 10.1016/j.jvir.2014.08.028 10.1016/j.jvir.2014.08.028 25311968

[8] Rezek I, Roberts S (1998) Envelope extraction via complex homomorphic filtering. Technical Report TR-98-9 Technical report

[9] Stadler A, Schima W, Ba’ssalamah A, Kettenbach J, Eisenhuber E (2007) Artifacts in body mr imaging: their appearance and how to eliminate them. Eur Radiol 17:1242–55. 10.1007/s00330-006-0470-4 17149625 10.1007/s00330-006-0470-4

[10] Reusz G, Langer C, Jakab L, Morvay Z (2012) Ultrasound-guided vascular access: the importance of the needle bevel. Can J Anesthesia/Journal canadien d’anesthésie 59:499–500 10.1007/s12630-012-9683-y 22395825

[11] Illanes A, Boese A, Friebe M, Hansen C (2020) Feasibility check: Can audio be a simple alternative to force-based feedback for needle guidance? In: International conference on medical image computing and computer-assisted intervention. https://api.semanticscholar.org/CorpusID:222136579

[12] Illanes A, Boese A, Maldonado Zambrano I, Pashazadeh A, Schaufler A, Navab N, Friebe M (2018) Novel clinical device tracking and tissue characterization using proximally placed audio signal acquisition and processing. Sci Rep. 10.1038/s41598-018-30641-0 30104613 10.1038/s41598-018-30641-0 PMC6089924

[13] Renna F, Illanes A, Oliveira J, Esmaeili N, Friebe M, Coimbra M (2019) Assessment of sound features for needle perforation event detection 2019:2597–2600. 10.1109/EMBC.2019.8857098 10.1109/EMBC.2019.8857098 31946428

[14] Schaufler A, Sühn T, Esmaeili N, Boese A, Wex C, Croner R, Friebe M, Illanes A (2019) Automatic differentiation between veress needle events in laparoscopic access using proximally attached audio signal characterization. Curr Direct Biomed Eng 5:369–371. 10.1515/cdbme-2019-0093

[15] Illanes A, Sühn T, Esmaeili N, Maldonado Zambrano I, Schaufler A, Chen C-H, Boese A, Friebe M (2019). Surgical audio guidance surag Extracting non-invasively meaningful guidance information during minimally invasive procedures. 10.1109/BIBE.2019.00108

[16] Al-Maatoq M, Boese A, Henke H-W, Friebe M (2019) Primary design concept for non-metallic needle for mri guided spinal applications, vol 2019. 10.1109/EMBC.2019.8856995 10.1109/EMBC.2019.8856995 31946291

[17] Sühn T, Esmaeili N, Mattepu SY, Spiller M, Boese A, Urrutia R, Poblete V, Hansen C, Lohmann CH, Illanes A et al (2023) Vibro-acoustic sensing of instrument interactions as a potential source of texture-related information in robotic palpation. Sensors 23(6):3141 36991854 10.3390/s23063141 PMC10056323

[18] Costa Junior JD, de Seixas JM, Miranda de Sá AMFL (2019) A template subtraction method for reducing electrocardiographic artifacts in emg signals of low intensity. Biomed Signal Process Control 47:380–386. 10.1016/j.bspc.2018.09.004

[19] Zhou P, Kuiken TA (2006) Eliminating cardiac contamination from myoelectric control signals developed by targeted muscle reinnervation. Physiol Meas 27(12):1311. 10.1088/0967-3334/27/12/005 10.1088/0967-3334/27/12/005 17135702

[20] He K, Zhang X, Ren S, Sun J (2016) Deep residual learning for image recognition. In: Proceedings of the IEEE conference on computer vision and pattern recognition, pp 770–778

[21] Seibold M, Maurer S, Hoch A, Zingg P, Farshad M, Navab N, Fürnstahl P (2021) Real-time acoustic sensing and artificial intelligence for error prevention in orthopedic surgery. Sci Rep 11(1):3993 33597615 10.1038/s41598-021-83506-4 PMC7889943

[22] Paszke A, Gross S, Massa F, Lerer A, Bradbury J, Chanan G, Killeen T, Lin Z, Gimelshein N, Antiga L et al (2019) Pytorch: an imperative style, high-performance deep learning library. Adv Neural Inf Process Syst 32

[23] Tulsiani S, Su H, Guibas LJ, Efros AA, Malik J (2017) Learning shape abstractions by assembling volumetric primitives. In: Proceedings of the IEEE conference on computer vision and pattern recognition, pp 2635–2643

[24] Stadler A, Schima W, Ba-Ssalamah A, Kettenbach J, Eisenhuber E (2007) Artifacts in body mr imaging: their appearance and how to eliminate them. Eur Radiol 17:1242–1255 17149625 10.1007/s00330-006-0470-4

